# Association between Serum Vitamin D and Irritable Bowel Syndrome Symptoms in a Sample of Adults

**DOI:** 10.3390/nu14194157

**Published:** 2022-10-06

**Authors:** Myriam Abboud, Suzan Haidar, Nadine Mahboub, Dimitrios Papandreou, Fatme Al Anouti, Rana Rizk

**Affiliations:** 1 Department of Health Sciences, College of Natural and Health Sciences, Zayed University (Dubai Campus), Dubai P.O. Box 19282, United Arab Emirates; 2 Department of Nutrition and Food Science, Faculty of Arts and Sciences, Lebanese International University, Beirut P.O. Box 146404, Lebanon; 3 Department of Health Promotion, School CAPHRI, Care and Public Health Research Institute, Faculty of Health, Medicine and Life Sciences, Maastricht University, 6200 MD Maastricht, The Netherlands; 4 Department of Health Sciences, College of Natural and Health Sciences, Zayed University (Abu Dhabi Campus), Abu Dhabi P.O. Box 144534, United Arab Emirates; 5 Department of Natural Sciences, School of Arts and Sciences, Lebanese American University, Byblos P.O. Box 36, Lebanon; 6 Institut National de Santé Publique, d’Epidémiologie Clinique, et de Toxicologie (INSPECT-LB), Beirut P.O. Box 14404, Lebanon

**Keywords:** irritable bowel syndrome, Vitamin D, Lebanon, adults

## Abstract

Vitamin D may be associated with irritable bowel syndrome (IBS) pathways. This cross-sectional study evaluated the associations between serum Vitamin D and IBS symptoms in a sample of Lebanese adults. Participants (*n* = 230; mean (SD) age: 43.36 (16.05) years, 62.9% females) were adults, free of diseases affecting Vitamin D metabolism, and recruited from a large university and the surrounding community. Serum Vitamin D (25-hydroxyvitamin D) was assessed using an automated chemiluminescence micro-particle immunoassay kit. The Birmingham IBS Symptom Questionnaire total scale, and pain, constipation, and diarrhea subscales were used to study IBS symptoms. Four linear regression analyses were performed, taking respectively the total scale and each of the subscales as the dependent variable. Vitamin D was forced into each model. Covariates included sociodemographic and medical variables, fluid intake, physical activity, sleep quality, stress, and adherence to the Mediterranean diet. Mean (SD) serum Vitamin D was 17.53 (12.40) ng/mL and mean (SD) Birmingham IBS Symptom Questionnaire was 16.98 (15.16) (pain: 20.75 (23.63), constipation: 25.06 (29.99), diarrhea: 9.88 (13.37)). Serum Vitamin D was not associated with the total score, nor with any of the subscales (*p* > 0.05 for the four regression analyses). Serum Vitamin D was not associated with IBS symptoms in a sample of Lebanese adults, adding to the controversy in this field. Further understanding of the pathophysiological mechanisms involved in Vitamin D and IBS is warranted.

## 1. Introduction

Irritable bowel syndrome (IBS) refers to a chronic gastrointestinal disorder marked by recurrently altered bowel function, urgency, abdominal distress, gas, and bloating with no detectable organic cause [1,2]. This chronic syndrome may be categorized into three subgroups according to international ROME-III consensus criteria: IBS-D with a predominance of diarrhea, IBS-C for constipation predominance, and IBS-M for the mixed type [3]. The pathological process of IBS is not yet fully understood, yet several factors have been acknowledged as interactional contributors [4]. These factors include intestinal damage, inflammatory disturbances, microbiota fluctuations, genetic features, and, most recently, psychological stress [5,6]. IBS is one of the most prevalent disorders, affecting around 1 in 10 people [7].

IBS has been associated with a cluster of negative consequences, including physical ailments such as abdominal pains, disturbed bowel movements, cramping, bloating, and sleep disturbances [8,9,10]; psychological problems such as increased anxiety, depression, stress, frustration, and shame; social impacts including compromised social activities, strained relationships, avoidance, and isolation; as well as economic repercussions with a negative impact on work productivity [11]. In addition to its effect on the individual, IBS substantially affects societies with the increased utilization of health resources and associated costs [12].

Given the high burden of IBS [11], the need to better understand its pathological processes informs better treatment strategies. Vitamin D deficiency is thought to be strongly linked to several systemic diseases [13,14]. It is involved in bone remodeling, the regulation of calcium absorption in the intestines, and intervening in cellular mechanisms [15,16]. Moreover, Vitamin D was shown to interfere with an immune bacterial response, antigen presentation, and immunity regulation [17,18,19], all of which propose implications for IBS pathways [20]. Furthermore, several studies have suggested that patients with IBS tend to have low Vitamin D levels [21,22,23].

The pathophysiology of IBS is multifactorial and complex, and risk factors such as genetics, diet, and the microbiome operate differently across ethnicities and geographical locations. Evidence from various settings is needed to better shape our understanding of this disorder and strategies to manage it [7]. The present study aims to evaluate the associations between serum Vitamin D and IBS symptoms in a sample of Lebanese adults while adjusting for potential confounders, including sociodemographics, weight status, sleep, stress, physical activity, eating pattern, and fluid intake.

## 2. Materials and Methods

### 2.1. Design

This was a cross-sectional study.

### 2.2. Subjects

Lebanese adults were invited to participate in this study through community announcements. Participants were asked to come to the data collection clinic fasting for at least 8 h and were included in the study if they met the following inclusion criteria: aged between 18 and 65 years, of Lebanese nationality, free of active infections (including COVID-19), not pregnant or lactating, do not use medications that affect the metabolism of Vitamin D such as seizure drugs: Phenobarbital and Dilantin (phenytoin), and anti-tuberculosis drugs, and free of any preexisting specified disease that affects the metabolism of Vitamin D such as significant renal or liver disease. 

### 2.3. Ethical Considerations

The participants were informed about the study objectives, protocol, and the right to withdraw from the study at any time and were only included in the study once they gave verbal consent. Ethical approval was obtained from the Lebanese International University’s Institutional Review Board (IRB) (case number: LIUIRB-220201-SH-111).

### 2.4. Data Collection

#### 2.4.1. Blood withdrawal

A 5-mL blood sample was then collected into a sterile serum separator tube with a clot activator by a certified phlebotomist. Blood samples were then transported via a thermally insulated box to the laboratory where samples were centrifuged at 4000 revolutions per minute for 10 min and analyzed for serum 25 hydroxyvitamin D (ng/mL) using an automated chemiluminescence micro-particle immunoassay (CMIA) kit (ARCHITECT; Abbott Laboratories, Abbott Park, IL, USA). This is a delayed one-step immunoassay including a sample pre-treatment for the quantitative determination of Vitamin D in competitive CMIA technology with flexible assay protocols.

#### 2.4.2. Questionnaires

Demographic and medical history questionnaire: included several questions related to age, gender, educational level, employment status, socioeconomic status, smoking status, and personal and family history of chronic diseases.

Birmingham IBS scale: this is a self-administered 11-item symptom questionnaire (based on Rome II criteria). Questions assess IBS-related symptoms in the previous four weeks, whereby each question has a standard response scale. Symptoms are measured based on a 6-point Likert scale (0–5) ranging from all of the time to none of the time and converted to 100. The scale has 3 dimensions (pain, constipation, and diarrhea) and is designed to enable assessment of symptom burden. In the validation study by Roalfe et al. [24], the score had a high internal validity (Cronbach’s α of 0.74 for pain, 0.79 for constipation, and 0.90 for diarrhea), as well as good external validity (r = −0.3 to −0.6) for pain and diarrhea and moderate external validity (r = −0.2 to −0.3) for constipation; with all dimensions being reproducible (ICCs 0.75 to 0.81).

Mediterranean Diet Adherence Screener (MEDAS): this is a 14-item questionnaire adapted from the PREvencion con DIetaMEDiterranea (PREDIMED) [25] study. The questions are related to food intake/frequency of foods. Answers that are in favor of the Mediterranean date are scored with one point, whereas unfavorable responses are given a score of 0. The final score is calculated by adding all responses to the 14 questions. The final score ranges between 0 and 14, whereby higher scores indicate more adherence to the Mediterranean diet. MEDAS showed to be a valid tool for rapidly assessing and providing advice on adherence to the Mediterranean diet compared with an extensive full-length food frequency questionnaire [26]. MEDAS has been extensively used in the international literature and was validated for various settings and populations.

The International Physical Activity Questionnaire (IPAQ) Short Form [27]: the validated Arabic version of the questionnaire was used [28]. IPAQ-Short Form includes seven questions regarding duration and frequency of light, moderate, and vigorous physical activity performed in the past week. The Metabolic Equivalent of Tasks (METs) are calculated by multiplying the total minutes expended in a certain activity by the frequency (days) by the constants of 3.3, 4.0, and 8.0 for light, moderate, and vigorous activity, respectively. The total MET values are calculated by totaling the respective MET values for all activities that were performed in bouts that were more than 10 minutes in duration.

The Pittsburgh Sleep Quality Index (PSQI): this questionnaire consists of 9 questions, four of which assess sleep duration (hours), duration needed to fall asleep, amount of time required to wake up, and time spent in bed while awake. The five other questions assess reasons for sleep troubles. A total score is computed using an algorithm adapted from the developers of the questionnaire. Higher scores (≥5) indicate poor sleep quality [29]. The Arabic version of the questionnaire, culturally adapted by Haidar et al. [30], was used.

The 10-item Cohen Perceived Stress Scale (PSS-10): this is a 10-item questionnaire aimed at measuring stress levels in the last month. Questions investigate feelings for which respondents find their present life situation unpredictable, uncontrollable, and stressful. PSS uses a 5-point scale ranging from never (0) to very often (4). The total score ranges from 0 to 40, where higher scores indicate higher perceived stress levels [31,32]. The Arabic version of the questionnaire, validated by Chaaya et al. [33], was used.

The Brief Questionnaire to Assess Habitual Beverage Intake Questionnaire (BEVQ): this is a brief food frequency questionnaire used to rapidly assess habitual beverage intake among adults in the previous month, and to determine possible associations of beverage consumption with health-related outcomes. Patients were asked to indicate the type, frequency, and number of beverages consumed. Total fluid intake (fl oz) and fluid intake of sugar-sweetened beverages (fl oz) were computed for this study [34].

The Birmingham IBS scale, MEDAS, and BEVQ were translated into Arabic following best practices [35]. First, the original tools were translated into Arabic, then the Arabic version was translated back into English. The two English versions were compared, and essential adjustments were made to the Arabic version.

The questionnaire was pilot-tested on ten adults prior to data collection. Feedback from the pilot was used to produce the final version of the questionnaire.

### 2.5. Statistical Analysis

The data were analyzed using SPSS, version 25. A descriptive analysis was done using the counts and percentages for categorical variables and mean and standard deviation for continuous measures. Normality distribution was checked using visual inspection of the histogram and verified by checking the normality line of the regression plot and the scatter plot of the residual. Independent-sample *t*-test was used to compare the mean of the Birmingham IBS symptom questionnaire and subscales (pain, constipation, and diarrhea) between two groups, whereas ANOVA test was used to compare three or more means. Pearson correlation test was used to evaluate the association between continuous variables and the Birmingham IBS symptom questionnaire and each of the subscales (pain, constipation, and diarrhea). Four multivariable linear regression analyses using the Enter method were performed, taking respectively the Birmingham IBS symptom questionnaire total scale and each of the subscales (pain, constipation, and diarrhea) as the dependent variable and variables showing a *p*-value less than 0.2 in the bivariate analysis as independent variables, in addition to Vitamin D which was forced into each model. *p*-value less than 0.05 was considered significant.

## 3. Results

### 3.1. Demographics and Medical Characteristics

In total, 230 males and females participated in the study. The demographic and medical characteristics of the participants are presented in Table 1. More than half of the sample were females (62.9%), married (55.7%), and with low monthly income (50.5%). The mean age of the participants was 43.36 ± 16.05 years, and the mean body mass index (BMI) was 28.43 ± 6.10 kg/m^2^. Almost half of the participants had a university level of education (46.2%) and were employed at the time of the data collection (47.0%). Around a quarter (21%) were cigarette smokers, and 31.6% smoked waterpipe. In addition, 18.1% of the participants had diabetes mellitus (type 1 or type 2), 28.5% had lipid metabolism disorders, and 20.8% had hypertension. The mean serum Vitamin D of the sample was 17.53 ± 12.40 ng/mL; 67.4% of the participants had a Vitamin D serum level ≤ 20 ng/mL.

### 3.2. Description of the Scales Used

The median, mean, standard deviation, and range of the scales used in this study are described in Table 2. The mean IBS total scale, IBS pain subscale, IBS constipation subscale, and IBS diarrhea subscale were 16.98 ± 15.16, 20.75 ± 23.63, 25.06 ± 29.99, and 9.88 ± 13.37, respectively.

### 3.3. Bivariate Analysis

The results of the bivariate analysis taking the Birmingham IBS total scale and subscales as dependent variables are displayed in Table 3. A significantly higher mean IBS total scale (M: 11.57 ± 14.09; F: 20.18 ± 14.90; *p* < 0.001), IBS pain subscale (M: 14.71 ± 19.67; F: 24.31 ± 25.07; *p* = 0.002), IBS constipation subscale (M: 15.36 ± 23.90; F: 30.79 ± 31.77; *p* < 0.001), and IBS diarrhea subscale (M: 7.41 ± 13.21; F: 11.33 ± 13.30; *p* = 0.035) were found among females as compared with males. Moreover, the mean IBS pain subscale (41.21 ± 26.30; *p* = 0.022) and IBS diarrhea subscale (14.50 ± 16.52; *p* = 0.047) were significantly higher among illiterate participants and those having diabetes, respectively. 

A significant negative correlation was found between the MEDAS and IBS pain subscale (r = −0.59; *p* = 0.018). On the other hand, a significant positive correlation was found between the PSQI scale and the IBS total scale (r = 0.253; *p* < 0.001), IBS pain subscale (r = 0.191; *p* = 0.004), IBS constipation subscale (r = 0.003; *p* = 0.004), and IBS diarrhea subscale (r = 0.163; *p* = 0.015). Moreover, the PSS was positively correlated with the IBS total scale (r = 0.213; *p* = 0.001), IBS pain subscale (r = 0.181; *p* = 0.007), and IBS constipation subscale (r = 0.213, *p* = 0.001). Finally, the BMI was positively correlated with the IBS total scale (r = 0.148; *p* = 0.028), IBS pain subscale (r = 0.189; *p* = 0.005), and IBS diarrhea subscale (r = 0.183; *p* = 0.006).

### 3.4. Multivariable Analysis

Table 4 illustrates four linear regressions taking the IBS total scale and each of the IBS subscales as dependent variables. Male gender was significantly associated with lower IBS total scale (Beta = −5.27, *p* = 0.019, 95% CI: −9.685, −0.867). Higher BMI was significantly associated with higher IBS pain subscale (Beta = 0.669, *p* = 0.031, 95% CI: 0.061,1.276). Primary education level and higher MEDAS scale were significantly associated with lower IBS pain subscale, respectively (Beta = −20.37, *p* = 0.02, 95% CI: −37.509, −3.237; Beta = −1.83, *p* = 0.021, 95% CI: −3.391, −0.280). Furthermore, male gender was significantly associated with a lower IBS constipation scale (Beta = −12.54, *p* = 0.006, 95% CI: −21.466, −3.622). No significant association was found between the variables and the IBS diarrhea subscale (*p* > 0.05). Vitamin D was not associated with the IBS total scale nor any of its subscales (*p* > 0.05). 

## 4. Discussion

Our results suggest that serum Vitamin D is not associated with IBS symptoms in a sample of Lebanese adults. This finding adds to the growing controversy in this field. Although our findings are in contrast to previous studies highlighting Vitamin D’s role in the pathogenicity of IBS, owing to its function in the intestinal barrier and mucosal inflammatory state [17,18,19,23,36,37], and to other research reporting alleviation in gastrointestinal symptoms with Vitamin D supplementation [38], they are parallel to those reported by Williams et al. [39], who found no benefit on gastrointestinal disturbances following Vitamin D supplementation even though baseline deficiencies were adjusted. The variability of our results could be attributed to confounding variables such as Vitamin D supplementation and body fat composition [39]; or to the established IBS syndrome heterogeneity [39]. Moreover, Vitamin D deficiencies among IBS individuals may be attributed to diet and lifestyle deviations to avoid symptoms triggering thus limiting exposures to Vitamin D sources such as off-putting outdoor activities or restraining from certain meals [40]. Furthermore, Agnello et al. [41] supported a connection for microbiome composition changes in IBS pathogenesis, suggesting clinical relevance in monitoring and investigating the microbiome in patients with IBS [41]. Due to its heterogeneous nature, there may be a cumulative alteration in the gut microbiome leading to dysbiosis and increased risk of chronic gastroenterological conditions. Future large studies are needed to better understand the conditions for IBS pathogenesis and its association with risk factors.

Our findings demonstrate that females had a higher propensity for experiencing IBS symptoms than males, specifically for constipation. This could be attributed to differences in intestinal motor and sensory functions, hormones, and microbiota between the genders linked to gut-brain interactions [42,43]. It is well known that estrogen and progesterone hormones inhibit smooth muscle contraction, which has an effect on peristalsis [42] and thus constipation. Additionally, sex hormones have an impact on bacterial growth, expression, and metabolism which can explain the differences between genders [42]. The exact mechanisms still need to be elucidated.

Additionally, our results reveal that a higher BMI is associated with increased IBS pain symptoms; however, we did not find an association with total IBS scores. These findings are in line with other studies reporting increased IBS symptoms in obese participants [44]. Furthermore, Clements et al. [45] found that in obese subjects, IBS symptoms decreased secondary to bariatric surgery. One possible mechanism where IBS symptoms develop in obese persons is related to the altered small bowel and colonic transit time [46]. Given the limited data with regard to altered intestinal motility in obesity, additional investigation is warranted before this can be evoked as an explanation for the development of IBS symptoms among this population group. Moreover, low-fiber and high refined-carbohydrate diets are linked to obesity and are another potential contributor to IBS symptoms [47]. Finally, gut microbiota shift, reported in both obesity and IBS, may also explain the possible connection [48]. The above mechanisms are still insufficient to establish a causal relationship between gut microbiota shifts and IBS symptoms in obese patients; however, the main question lies in whether IBS symptoms are likely to increase obesity or whether it is the other way around. Future studies should further address this question. 

Another interesting finding in our study is the lack of association between stress and any of the IBS dimensions studied. This is in contrast to an abundance of studies that have reported higher gastrointestinal disturbances associated with psychological distress [49,50]. Surdea-Blaga et al. [51], found that stressful life events may aggravate symptoms of IBS in approximately one-third of patients with IBS. These conflicting results could be attributed to genetic and ethnic factors [52]. Tran [53] suggests that research on social stress should take ethnic identification and gender variables into consideration. The direction of the connection between stress and IBS also needs further research. IBS diagnosis can be a significant predictor of perceived stress, and higher perceived stress increases the odds of IBS diagnosis [54]. 

Interestingly, our results show that adherence to the Mediterranean diet was inversely associated with the IBS pain subscale. This was unexpected since IBS dietary recommendations suggest that a diet low in fermentable oligosaccharides, disaccharides, monosaccharides, and polyols (FODMAP) is used to manage IBS symptoms. However, it is possible that the anti-inflammatory properties of the Mediterranean diet helped in pain management among IBS patients [55]. 

Our study presented several strengths. It is the first study of its kind to be carried out in the Middle East assessing Vitamin D levels as related to IBS symptoms while accounting for possible confounders. Furthermore, biochemical parameters were analyzed in a certified laboratory in Lebanon. Additionally, we used validated assessment tools and scales. In contrast, the study presents some limitations. First, the study has a cross-sectional design not allowing casual inferences [56]. Furthermore, as for the general Lebanese population [57], the majority of the sample had Vitamin D deficiency, which could have confounded or further underestimated any association with IBS symptoms. Moreover, the small sample size might have been insufficient to detect significant associations between Vitamin D and symptoms of IBS. Finally, this study was carried out during an unpreceded economic crisis, which could have probably heightened the stress levels of the participants and hindered our ability to find any association between stress and IBS.

## 5. Conclusions

This study highlighted the association of gender, BMI, and diet with IBS symptoms, whereas the relationship with physical activity, psychological distress, and Vitamin D could not be established. Further understanding of the pathophysiological mechanisms involved in Vitamin D and IBS is necessary to better uncover their association and to develop approaches that may be effective for IBS patients.

## Figures and Tables

**Table 1:** Sociodemographic and other characteristics of the participants (*N* = 230).

Variable	*N* (%)
**Gender**	
Male	82 (37.1%)
Female	139 (62.9%)
**Marital status**	
Single/widowed/divorced	98 (44.3%)
Married	123 (55.7%)
**Education level**	
University degree	102 (46.2%)
High school	41 (18.6%)
Middle education	37 (16.7%)
Primary education	30 (13.6%)
Illiterate	11 (5.0%)
**Socioeconomic status**	
Low	111 (50.5%)
Medium	102 (46.4%)
High	7 (3.2%)
**Profession**	
Yes	103 (47.0%)
No	116 (53.0%)
**Cigarette smoking**	
Never	158 (71.8%)
Previous smoker	16 (7.3%)
Mild (1–10 cigarettes/day)	20 (9.1%)
Moderate (11–20 cigarette/day)	14 (6.4%)
Heavy smoking (>20 cigarettes/day)	12 (5.5%)
**Waterpipe smoking**	
Never	129 (58.4%)
Previous smoker	22 (10.0%)
Mild (≤1/day)	60 (27.1%)
Moderate (>1/day)	10 (4.5%)
**Having Type 1 diabetes or type 2**	
No	172 (77.8%)
Yes, type 1	3 (1.4%)
Yes, type 2	33 (14.9%)
Yes, I do not know	4 (1.8%)
Do not know	9 (4.1%)
**Taking diabetes medication**	
Yes	33 (71.7%)
No	13 (28.3%)
**Family history of diabetes**	
No	84 (38.2%)
Yes, type 1	16 (7.3%)
Yes, type 2	73 (33.2%)
Yes, I do not know	27 (12.3%)
Do not know	16 (7.3%)
Type 1 and type 2	4 (1.8%)
**Having disorders of lipid metabolism**	
Yes	63 (28.5%)
No	129 (58.4%)
Do not know	29 (13.1%)
**Taking medication for disorders of lipid metabolism**	
Yes	44 (47.8%)
No	48 (52.2%)
**History of disorders of lipid metabolism in the family**	
Yes	85 (38.6%)
No	105 (47.7%)
Do not know	30 (13.6%)
**Having hypertension**	
Yes	46 (20.8%)
No	164 (74.2%)
Do not know	11 (5.0%)
**Taking medication for hypertension**	
Yes	41 (70.7%)
No	17 (29.3%)
**Having hypertension in the family**	
Yes	126 (57.3%)
No	76 (34.5%)
Do not know	18 (8.2%)
	**Mean ± SD**
Age	43.36 ± 16.05
Body mass index (kg/m^2^)	28.43 ± 6.10

**Table 2:** Description of the used scales.

	Mean (SD)	Median	Minimum	Maximum
Birmingham IBS symptom questionnaire	16.98 (15.16)	14.54	0	100
IBS Pain	20.75 (23.63)	13.33	0	100
IBS Constipation	25.06 (29.99)	13.33	0	100
IBS Diarrhea	9.88 (13.37)	4.00	0	100
PSQI	6.99 (3.63)	6.00	0	17.00
PSS	19.84 (7.32)	20.00	0	40.00
IPAQ total (Log10)	3.15 (0.49)	3.19	2.00	4.12
MEDAS	5.98 (2.17)	6.00	0	20.00
BEVQ total	49.69 (25.22)	47.43	2.86	145.14
BEVQ Sugar-sweetened beverages	13.52 (14.52)	8.57	0	73.14
BEVQ water	24.55 (17.69)	24.00	0	96.00

BEVQ: Brief Questionnaire to Assess Habitual Beverage Intake, IBS: Irritable Bowel Syndrome, IPAQ: International Physical Activity Questionnaires, PSQI: Pittsburgh Sleep Quality Index, PSS: Perceived Stress Scale, MEDAS: Mediterranean Diet Adherence Score.

**Table 3:** Bivariate analysis taking the IBS total and subscores as the dependent variables.

	IBS Total	IBS Pain	IBS Constipation	IBS Diarrhea
	Mean ± SD	Mean ± SD	Mean ± SD	Mean ± SD
**Gender**				
Male	11.57 ± 14.09	14.71 ± 19.67	15.36 ± 23.90	7.41 ± 13.21
Female	20.18 ± 14.90	24.31 ± 25.07	30.79 ± 31.77	11.33 ± 13.30
*p*-value	**<0.001**	**0.002**	**<0.001**	**0.035**
**Marital status**				
Single/widowed/divorced	18.51 ± 16.47	22.99 ± 26.21	26.93 ± 29.56	10.77 ± 14.05
Married	15.77 ± 13.97	18.97 ± 21.28	23.57 ± 30.36	9.17 ± 12.83
*p*-value	0.182	0.220	0.409	0.377
**Education level**				
University degree	16.77 ± 17.09	18.95 ± 21.81	24.05 ± 29.50	11.09 ± 15.88
High school	17.78 ± 13.43	23.57 ± 26.03	27.47 ± 30.33	8.48 ± 9.96
Middle education	16.65 ± 13.04	21.26 ± 27.43	24.86 ± 28.57	8.97 ± 11.44
Primary education	13.15 ± 11.40	14.88 ± 15.91	20.66 ± 31.38	7.60 ± 9.87
Illiterate	27.60 ± 14.96	41.21 ± 26.30	38.18 ± 35.16	13.09 ± 13.51
*p*-value	0.112	**0.022**	0.539	0.567
**Socioeconomic status**				
Low	18.24 ± 16.20	22.58 ± 23.20	25.40 ± 30.96	11.35 ± 15.72
Medium	15.47 ± 14.14	19.21 ± 24.63	23.46 ± 28.38	8.43 ± 10.48
High	15.06 ± 6.61	13.33 ± 14.90	32.38 ± 28.13	5.71 ± 7.25
*p*-value	0.388	0.413	0.703	0.201
**Profession**				
Yes	15.88 ± 16.39	20.45 ± 23.47	22.13 ± 29.30	9.39 ± 15.51
No	17.96 ± 14.11	21.03 ± 24.00	27.75 ± 30.60	10.24 ± 11.16
*p*-value	0.315	0.857	0.168	0.642
**Cigarette smoking**				
Never	15.39 ± 13.86	18.86 ± 21.48	22.70 ± 28.32	8.93 ± 12.08
Previous smoker	20.79 ± 14.51	22.08 ± 30.18	37.08 ± 38.41	10.25 ± 11.59
Mild (1–10 cigarettes/day)	20.81 ± 16.20	29.00 ± 26.95	27.66 ± 30.60	11.80 ± 11.85
Moderate (11–20 cigarette/day)	20.38 ± 14.70	23.33 ± 29.08	30.95 ± 30.67	12.28 ± 14.33
Heavy smoking (>20 cigarettes/day)	21.21 ± 26.78	25.00 ± 28.58	26.11 ± 36.56	16.00 ± 27.55
*p*-value	0.227	0.401	0.374	0.388
**Waterpipe smoking**				
Never	17.67 ± 16.30	19.94 ± 22.38	28.21 ± 32.06	9.98 ± 13.78
Previous smoker	14.13 ± 10.64	20.90 ± 23.12	17.27 ± 26.87	8.18 ± 8.13
Mild (≤1/day)	16.42 ± 14.24	20.33 ± 24.53	21.66 ± 26.79	10.93 ± 14.72
Moderate (>1/day)	17.81 ± 14.76	33.33 ± 33.84	22.00 ± 24.35	6.00 ± 8.05
*p*-value	0.766	0.393	0.287	0.668
**Personal history of diabetes**				
Yes	16.72 ± 13.46	16.16 ± 19.73	21.00 ± 30.40	14.50 ± 16.52
No	17.04 ± 15.54	21.76 ± 24.33	25.96 ± 29.90	8.86 ± 12.40
*p*-value	0.904	0.175	0.344	**0.047**
	**Correlation coefficient**	**Correlation coefficient**	**Correlation coefficient**	**Correlation coefficient**
Vitamin D	−0.010	−0.004	−0.031	0.020
*p*-value	0.877	0.951	0.648	0.769
Age	−0.086	0.021	0.054	−0.003
*p*-value	0.205	0.754	0.425	0.961
BMI	0.148	0.189	−0.012	0.183
*p*-value	**0.028**	**0.005**	0.861	**0.006**
PSQI	0.253	0.191	0.197	0.163
*p*-value	**<0.001**	**0.004**	**0.003**	**0.015**
PSS	0.213	0.181	0.213	0.052
*p*-value	**0.001**	**0.007**	**0.001**	0.438
IPAQ total (Log10)	0.089	0.108	0.014	0.086
*p*-value	0.224	0.138	0.845	0.242
MEDAS	−0.101	−0.159	−0.050	−0.016
*p*-value	0.135	**0.018**	0.463	0.809
BEVQ total	−0.041	−0.050	−0.040	0.006
*p*-value	0.548	0.455	0.555	0.929
BEVQ Sugar-sweetened beverages	0.037	0.056	0.039	−0.020
*p*-value	0.586	0.411	0.563	0.772
BEVQ water	−0.119	−0.104	−0.085	−0.072
*p*-value	0.078	0.124	0.206	0.290

*p*-values marked in bold are <0.05; BEVQ: Brief Questionnaire to Assess Habitual Beverage Intake, BMI: Body Mass Index, IBS: Irritable Bowel Syndrome, IPAQ: International Physical Activity Questionnaires, PSQI: Pittsburgh Sleep Quality Index, PSS: Perceived Stress Scale, MEDAS: Mediterranean Diet Adherence Score.

**Table 4:** Multivariable linear regression analyses.

	Unstandardized Beta	Standardized Beta	*p*-Value	Confidence Interval	
				Lower	Upper
IBS Total					
Gender (Male vs. Female *)	−5.276	−0.168	**0.019**	−9.685	−0.867
Marital status (Married vs. single *)	−1.925	−0.063	0.375	−6.190	2.340
Education primary	−8.066	−0.183	0.120	−18.266	2.133
Education elementary	−6.505	−0.161	0.199	−16.460	3.450
Education secondary	−4.674	−0.120	0.351	−14.532	5.183
Education university	−3.417	−0.113	0.482	−12.986	6.153
MEDAS	−0.730	−0.105	0.113	−1.633	0.173
PSQI	0.582	0.140	0.058	−0.020	1.184
PSS	0.242	0.117	0.106	−0.051	0.535
BEVQ water	−0.040	−0.046	0.489	−0.153	0.073
BMI	0.285	0.115	0.096	−0.051	0.622
Vitamin D	−0.022	−0.018	0.784	−0.182	0.137
IBS Pain					
Gender (Male vs. Female *)	−4.272	−0.087	0.245	−11.499	2.955
Education primary	−20.370	−0.291	**0.020**	−37.509	−3.231
Education elementary	−13.503	−0.211	0.111	−30.155	3.149
Education secondary	−9.023	−0.144	0.283	−25.573	7.527
Education university	−11.178	−0.235	0.165	−26.989	4.633
MEDAS	−1.836	−0.166	**0.021**	−3.391	−0.280
PSQI	0.519	0.077	0.329	−0.527	1.565
PSS	0.337	0.102	0.188	−0.166	0.840
BEVQ water	−0.088	−0.068	0.353	−0.275	0.099
BMI	0.669	0.162	**0.031**	0.061	1.276
Personal history of diabetes (Yes vs. No *)	−7.922	−0.125	0.093	−17.177	1.333
IPAQ (log 10)	3.591	0.075	0.289	−3.075	10.256
Vitamin D	0.035	0.018	0.798	−0.232	0.301
IBS Constipation					
Gender (Male vs. Female *)	−12.544	−0.202	**0.006**	−21.466	−3.622
PSQI	0.918	0.111	0.144	−0.316	2.152
PSS	0.483	0.118	0.111	−0.111	1.077
BEVQ water	−0.035	−0.021	0.761	−0.262	0.192
BMI	−0.306	−0.062	0.352	−0.954	0.341
Vitamin D	−0.101	−0.042	0.524	−0.415	0.212
Profession (Yes vs. No *)	0.264	0.004	0.950	−8.049	8.577
IBS Diarrhea					
Gender (Male vs. Female *)	−2.508	−0.091	0.194	−6.302	1.285
PSQI	0.350	0.095	0.172	−0.154	0.854
BEVQ water	−0.044	−0.058	0.397	−0.145	0.058
BMI	0.277	0.127	0.069	−0.021	0.576
Vitamin D	0.019	0.017	0.793	−0.122	0.159
Personal history of diabetes (Yes vs. No *)	4.208	−0.091	0.078	−0.475	8.892

* Reference group. *p*-values marked in bold are <0.05. BEVQ: Brief Questionnaire to Assess Habitual Beverage Intake, BMI: Body Mass Index, IBS: Irritable Bowel Syndrome, IPAQ: International Physical Activity Questionnaires, PSQI: Pittsburgh Sleep Quality Index, PSS: Perceived Stress Scale, MEDAS: Mediterranean Diet Adherence Score.

## Data Availability

The data presented in this study are available on request from the corresponding author.
